# An Exploration of Barriers to Insulin Initiation for Physicians in Japan: Findings from the Diabetes Attitudes, Wishes and Needs (DAWN) JAPAN Study

**DOI:** 10.1371/journal.pone.0036361

**Published:** 2012-06-14

**Authors:** Hitoshi Ishii, Yasuhiko Iwamoto, Naoko Tajima

**Affiliations:** 1 Department of Endocrinology, Tenri Hospital, Tenri, Nara, Japan; 2 Tokyo Women’s Medical University, Shinjuku-ku, Tokyo, Japan; 3 Jikei University School of Medicine, Minato-ku, Tokyo, Japan; University of Michigan Medical School, United States of America

## Abstract

**Objective:**

Insulin is recommended as an appropriate treatment in type 2 diabetes patients with suboptimal glycemic control; however, its initiation is often delayed. We therefore conducted the DAWN (Diabetes Attitudes, Wishes and Needs) JAPAN study in an attempt to identify specific patient- and physician-related factors which contribute to delay of insulin initiation among Japanese patients with diabetes. In this report, we explored barriers for physicians which prevent timely insulin initiation.

**Methods:**

The DAWN JAPAN study is a multicenter, questionnaire-based survey, conducted between 2004 and 2005. Participating physicians were categorized as follows based on their expertise: Japan Diabetes Society (JDS) certified specialists (n = 77), JDS-affiliated physicians (n = 30), and non-JDS-affiliated physicians (n = 27). To assess physician barriers to insulin initiation, we have used a newly developed 27- item questionnaire.

**Results:**

The mean age of patients (n = 11,656) treated by participating physicians was 64.1 years. The mean duration of diabetes was 121.6 months, and their mean HbA1c was 7.5%. Insulin was used in 27.4% of total patients. With regard to physician barriers to insulin initiation, the biggest differences in concerns expressed by JDS-certified specialists and non-JDS-affiliated physicians were observed in the following items with statistical significance: “I do not have staff (nurse, pharmacists) who can assist with explanations” (1.3% vs 55.5%, respectively), “I have concerns about the use of insulin therapy in elderly patients” (38.1% vs 81.5%), and “It is difficult to provide guidance and education on insulin injection to patients” (16.9% vs 55.5%). The mean HbA1c at which physicians responded they would recommend insulin to their patients was 8.7%; however, they would reduce this level to 8.2% if they themselves required insulin.

**Conclusions:**

Our results demonstrated that physicians have concerns about insulin use, and suggested that their concerns can lead to delay of insulin initiation.

## Introduction

Strict glycemic control in type 2 diabetes mellitus (T2D) can prevent the onset and progression of diabetic complications [1], [2]. Nonetheless, achievement of recommended glycemic targets in patients with T2D in Japan remains less than optimal. According to the Diabetes Mellitus Treatment Guidelines published by the Japan Diabetes Society (JDS), an HbA_1c_ level of ≥8.4% (JDS) is considered “unacceptable” and represents the level at which treatment needs re-evaluating. Insulin treatment would be strongly recommended when HbA_1c_ ≤7.0% cannot be achieved with oral antidiabetic agents (OADs), but initiation of insulin is often delayed mainly due to patients’ hesitation to start insulin treatment [3]–[11] even in insufficient glycemic control [12].

The DAWN study, a cross-sectional international survey initiated in 2001 by Novo Nordisk in collaboration with the International Diabetes Federation, was conducted to identify a broad set of attitudes, wishes, and needs among both people with diabetes and care providers (physicians and nurses), consisting of more than 5,000 patients with diabetes and nearly 4,000 care providers in a total of 13 countries participated in [13]. The survey demonstrated that the most significant factor preventing the initiation of insulin therapy was patient resistance [13]–[15]. In addition, physician barriers to insulin initiation were demonstrated with the result that approximately 40% of physicians did not prefer to initiate insulin unless it became “absolutely necessary” [16] although most physicians recognized that insulin was an efficacious approach to the management of T2D [17].

On the basis of these key findings from the international DAWN study, we planned a series of surveys as the DAWN JAPAN study attempting to identify specific factors which contribute to delay of insulin initiation among both Japanese physicians and patients. As a better understanding of the barriers to insulin initiation both in physicians and patients is developed, more appropriate strategies can be implemented to encourage timely insulin initiation. This paper reports the survey results relevant to understanding physician barriers to insulin initiation in Japan.

## Methods

### Design and the Survey Process of the DAWN JAPAN Study

The DAWN JAPAN study is a multicenter, questionnaire-based survey, conducted between 2004 and 2005. Both physicians and patients participated in this study. The participating physicians were categorized based on the following: JDS-certified specialists, JDS-affiliated physicians, and non-JDS-affiliated physicians. The participating physicians of the each category were selected from throughout Japan roughly at a ratio of 2∶1∶1. As a patient sample, patients with T2D either treated with insulin or not, who were under care of participating physicians, were included in the survey.

**Figure 1:**
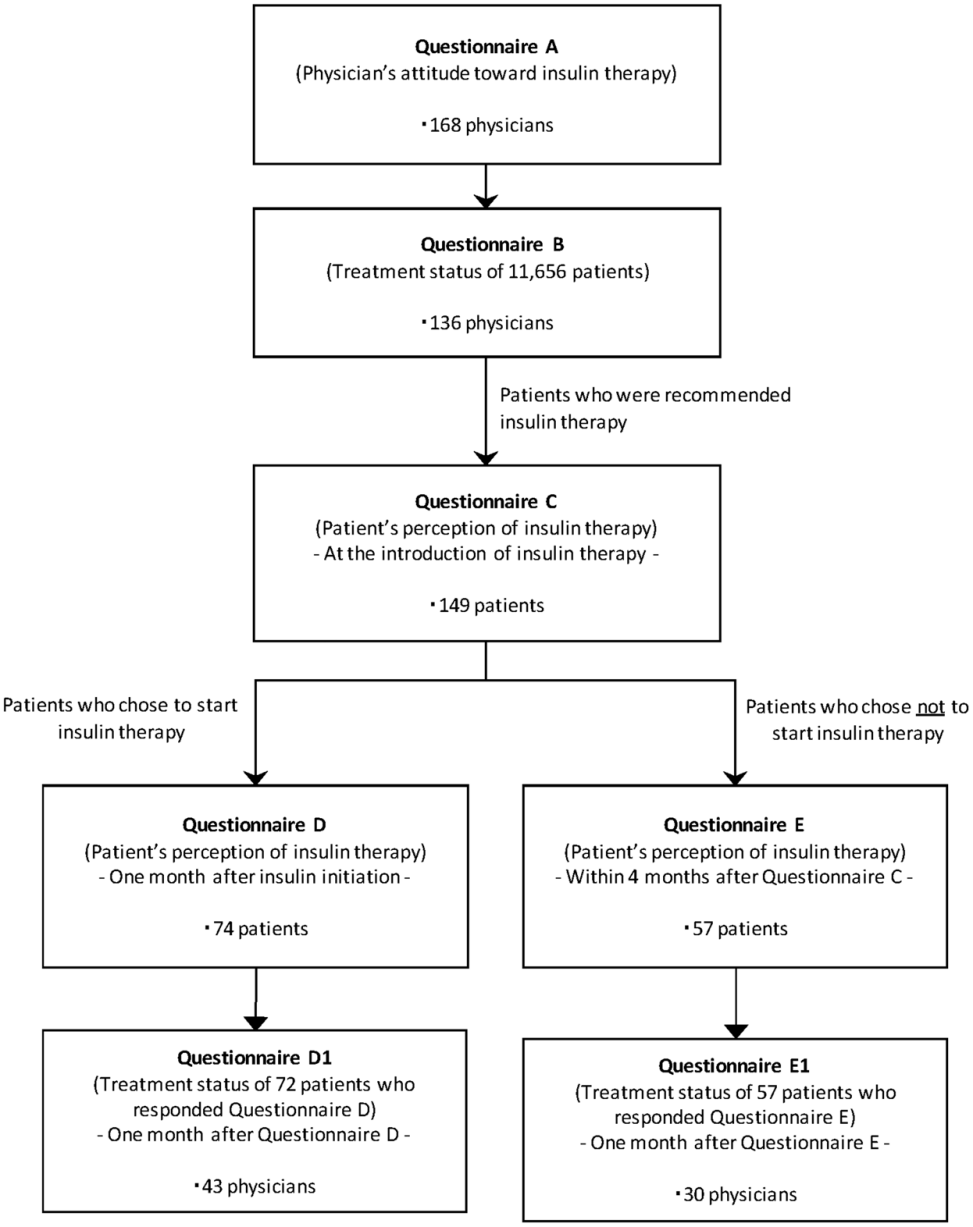
The survey process of DAWN JAPAN study.

The survey process of the DAWN JAPAN study is shown in Figure 1. Participating physicians first completed Questionnaire A, assessing their attitudes towards insulin as a treatment for T2D. The same physicians then completed Questionnaire B to obtain treatment status of the patients with T2D they treated during a 1-month period, up to a maximum of 100 patients. Of the patients whose treatment status was collected in Questionnaire B, those who were recommended to start insulin in the participating sites were asked to complete Questionnaire C, which examined their perceptions of insulin treatment. Of these patients, those who subsequently chose to start insulin treatment answered Questionnaire D a month after the start of insulin treatment to examine whether their attitudes toward to insulin would change after insulin had been initiated. Also, those who chose not to start insulin filled out Questionnaire E, 4 months after the end of Questionnaire B completion period to examine a change of their attitudes toward insulin treatment. The attending physicians filled out Question D1 or E1 to assess treatment status of their patients who completed either Questionnaire D or E a month after the completion of each questionnaire.

Due to the non-interventional nature of this study, the study protocol was approved centrally by the ethics committee of the DAWN JAPAN study group. The ethics committee of the DAWN JAPAN study group was responsible for the ethical and scientific quality of the study. The study was conducted in accordance with the Declaration of Helsinki [18] and with the Ethical Guidelines related to Epidemiological Research [19]. A written informed consent was obtained from all the participating patients in this survey.

### Assessment of Physician Barriers to Insulin Initiation

To assess physician barriers to insulin initiation, we developed 27-item questionnaire named the Physicians Attitude to Insulin Therapy questionnaire (PAINT), and included it in Questionnaire A. A five-point Likert scale (1. completely agree; 2. mostly agree; 3. neither agree nor disagree; 4. mostly disagree; 5. completely disagree) was employed in the PAINT. Content validity of the PAINT was assured based on review by DAWN JAPAN advisory panel and a pilot-test on a relevant sample of physicians. The psychometric properties of the PAINT were assessed by using the collected questionnaires in this survey. Internal consistency was excellent with cronbach α coefficient of 0.94. Exploratory cluster analysis demonstrated that the PAINT consisted of 5 clusters: “Issues with the doctor’s experience”, “Burden related to explanations”, “Consideration of burden on patients”, “Concerns regarding insulin therapy”, and “Concerns regarding hypoglycemia”. The second eigenvalues in each cluster were 0.81, 0.65, 0.71, 0.77, and 0.89, respectively. The proportion of total variation explained was 0.61.

In addition to the PAINT, the participating physicians were asked to answer their treatment strategies of T2D and HbA_1c_ level at which insulin initiation was recommended. Treatment status of their patients including currently receiving treatment, past diabetes history, and current HbA_1c_ were collected using Questionnaire B. The value for HbA_1c_ (%) is estimated as a National Glycohemoglobin Standardization Program (NGSP) equivalent value (%) calculated by the formula: HbA_1c_ (%)  =  HbA_1c_ (JDS value) +0.4, considering the relational expression of HbA_1c_ (JDS value) (%) measured by the previous Japanese standard substance and measurement methods and HbA1c [20].

### Statistical Analysis

The physicians who completed both the Questionnaire A and B were included into the analysis set for PAINT assessment. Descriptive analyses were performed on background data of physicians and their patients by the physician subgroups: JDS-certified specialists, JDS-affiliated physicians, and Non-JDS-affiliated physicians. In the analyses of the PAINT, a response distribution of each item was calculated, and the equality of distribution among the physician subgroups was analyzed using Kruskal–Wallis one-way analysis of variance by ranks test. A stepwise multiple linear regression analysis was conducted to explore which PAINT items are predictive of the rate of insulin use. The rate of insulin use was calculated by dividing the number of patients treated with insulin therapy by the number of patients with T2D treated in a month.

Statistical Package SAS Release 9.1 was used for the analyses. All statistical tests were two-tailed, and the level of statistical significance was set at 5%.

## Results

**Figure 2:**
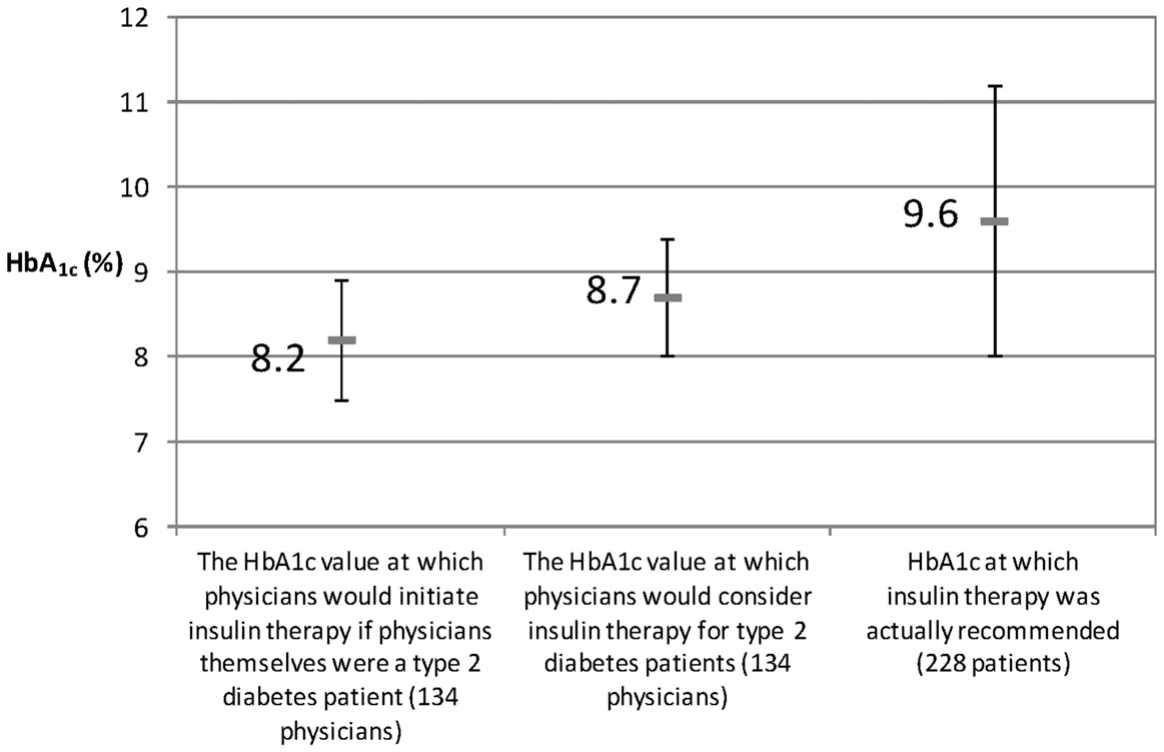
Gap between considered and actual recommended level of HbA1c. HbA_1c_ level at which insulin therapy would be recommended was compared to actual level at which insulin therapy was recommended (mean ± SD).

In total, 134 physicians completed both Questionnaire A and Questionnaire B (Table 1). JDS-certified specialists treated more patients per month than the other physician groups. Only 3.7% of physicians had no experience providing insulin therapy. Staff who is capable of providing guidance on diabetes treatment (nurses, pharmacists, or other staff) is present in 76.1% of the participating institutions in total, but 70.4% of non-JDS-affiliated physicians had no access to this support. The mean HbA1c at which physicians responded they would recommend insulin to their patients was 8.7±0.7% although if they themselves required insulin, the physicians responded they would reduce this level to 8.2±0.7% (Figure 2).

**Table 1:** Background information of participating physicians and their patients.

	Overall		Physician subgroup						P value
			JDS-certifiedspecialists		JDS-affiliatedphysicians		Non-JDS-affiliatedphysicians		
*Physician sample* (n, % ormean ±SD)	134		77	57.5%	30	22.4%	27	20.1%	
Age (years)	134	48.3±8.4	77	49.6±7.6	30	44.5±11.0	27	48.7±5.6	<0.0001†
Sex (male)	115	85.8%	65	84.4%	24	80.0%	26	96.3%	0.18382§
Type of work	115	85.8%	65	84.4%	24	80.0%	26	96.3%	0.18382§
Private practice	79	59.0%	37	48.1%	18	60.0%	24	88.9%	
Working at a hospital	55	41.0%	40	51.9%	12	40.0%	3	11.1%	
Number of patients withtype 2 diabetes per month	134	325.4±282.7	77	437.6±269.6	30	185.8±162.5	27	75.4±47.0	<0.0001†
Number of insulin-treatedpatients with type 2diabetes permonth	134	83.8±85.1	77	126.2±84.5	30	42.8±52.0	27	8.7±5.9	<0.0001†
Experience providing insulintherapy:									0.12928§
No	5	3.7%	1	1.3%	2	6.7%	2	7.4%	
Yes	129	96.3%	76	98.7%	28	93.3%	25	92.6%	
Staff capable of providingguidance on diabetestreatment:									<0.0001§
Absent	32	23.9%	4	5.2%	9	30.0%	19	70.4%	
Present	102	76.1%	73	94.8%	21	70.0%	8	29.6%	
Implementation of patienteducation (classrooms)regarding diabetes:									<0.0001§
Not implemented	40	30%	6	7.9%	12	40.0%	22	81.5%	
Irregularly implemented	24	18%	15	19.7%	6	20.0%	3	11.1%	
Regularly implemented	69	51.9%	55	72.4%	12	40.0%	2	7.4%	
The HbA1c value at whichI would consider insulintherapy for type 2diabetes patients	134	8.7±0.7	77	8.7±0.7	30	8.6±0.5	27	9.1±0.8	<0.0001†
The HbA1c value at whichI would initiate insulintherapy if I were a type 2diabetes patient	134	8.2±0.7	77	8.0±0.6	30	8.1±0.5	27	8.6±1.0	<0.0001†
*Patients treated by* *participating physicians*(n, % or mean ±SD)	11,656		7,403	64.6%	2,224	19.4%	1,829	16.0%	
Age (years)	11,621	64.1±11.8	7,384	63.2±11.8	2,218	65.6±11.6	1,821	65.9±11.1	<0.0001†
Sex (male)	6,344	54.9%	4,112	56.1%	1,166	52.8%	947	52.2%	0.00098§
HbA1c (%)	11,583	7.5±1.3	7,359	7.6±1.3	2,207	7.3±1.3	1,818	7.2±1.3	<0.0001†
BMI	11,391	24.2±3.8	7,193	24.1±3.8	2,198	24.4±3.9	1,802	24.4±3.8	<0.0001†
Duration of diabetes (months)	11,326	121.6±99.7	7,134	134.0±102.2	2,194	109.9±93.6	1,798	88.4±88.3	<0.0001†
Current Treatment:									<0.0001§
Diet and exercise only	2,071	17.8%	1,105	15.0%	431	19.4%	480	26.3%	
OADs only	6,378	54.8%	3,843	52.0%	1,274	57.4%	1,156	63.3%	
Insulin only	1,952	16.8%	1,545	20.9%	267	12.0%	112	6.1%	
OAD + insulin	1,235	10.6%	898	12.1%	247	11.1%	79	4.3%	

SD: standard deviation; P values were calculated by using chi-square test (§) or ANOVA (†).

The background information of 11,656 patients with T2D treated by the participating physicians was obtained from Questionnaire B (Table 1). Overall, mean HbA1c (±SD) was 7.5±1.3%. Regarding treatments being received, 17.8% of patients were receiving diet and exercise therapy only, 54.8% were using oral antidiabetic drugs (OADs) only, and 27.4% of patients were using insulin (alone or in combination with OADs). Of the patients treated by JDS-certified specialists, 33.0% were using insulin (alone or in combination with OADs), compared with 23.1% and 10.4% of patients treated by JDS-affiliated and non-JDS-affiliated physicians, respectively. Mean HbA_1c_ was 0.3–0.4 points higher in patients of JDS-certified specialists than in patients in other physician groups. The duration of diabetes was longer in patients of JDS-certified specialists than in other groups.

Of the patients surveyed in Questionnaire B, insulin treatment was recommended to 236 patients (2.0%) on the day they visited the hospital/clinic. The mean HbA_1c_ (±SD) in patients recommended insulin was 9.6±1.5% (Figure 2). Of patients with an HbA_1c_ ≥8.4% (n = 2,346; 20.1%), insulin was recommended to just 192 patients (8.2%).

### Physician Barriers (The Results of the PAINT)

The result of the PAINT is shown in Table 2. The percentage of expressing concerns relate to insulin (cumulative response of “completely agree” and “mostly agree”) were significantly different in the items among the physician groups except following items: “The patient would have to pay more for treatment” (P = 0.1576), “There is a higher risk of hypoglycemia with insulin therapy compared to other therapies” (P = 0.2168), and “Compliance with insulin therapy tends to be low” (P = 0.1799). The three most reported concerns were the same regardless of the expertise of the physician: “The patients would have to pay more for treatment” (76.6%, 53.3%, and 62.9%, for JDS-certified physicians, JDS-affiliated physicians, and non-JDS-affiliated physicians, respectively), “Patients would resist insulin therapy” (48.1%, 56.7%, 81.4%, respectively), and “I have concerns about the use of insulin therapy in elderly patients” (38.1%, 43.3%, 81.5%, respectively).

**Table 2:** Physician responses to concerns about insulin: result of PAINT.

Item	% respondents who answered “completely agree” and “mostly agree” in each item							P value†
	JDS-certified specialists (n = 77)	JDS-affiliatedphysicians (n = 30)			Non-JDS-affiliated physicians ( = 27)			
*Issues with the doctor’s experience*								
My reputation would suffer if I offered insulin therapy	0.0	6.7			7.4			0.0374
I’m not familiar with insulin therapy	2.6	16.6			37.0			<.0001
It is difficult to select the type of insulin and adjust the dose	3.9	16.7			37.0			<.0001
It is difficult to remember the many types of insulin preparations	5.2	10.0			25.9			<.0001
It is difficult to learn the methods of use of the many types of insulin injection devices (including insulin pens)	6.5	10.0			18.5			<.0001
It is difficult to learn new methods of insulin therapy	2.6	10.0			18.5			<.0001
My clinic/hospital is not equipped to provide insulin therapy	0.0	10.0			29.6			<.0001
In principle, I would rather avoid diabetes patients	1.3	0.0			0.0			<.0001
If necessary, I can refer the patient to a specialist	2.6	10.0			51.8			<.0001
*Burden related to explanations*								
It is time-consuming to explain injection methods and the use of injection devices	15.6	20.0			51.8			<.0002
It is time-consuming to explain hypoglycemia and its management	6.5	10.0			33.3			<.0001
I do not have staff (nurses, pharmacists) who can assist with explanations	1.3	16.7			55.5			<.0001
It is time-consuming to explain self-monitoring of blood glucose	14.5	10.0			34.6			0.0002
It is a hassle to purchase and manage the inventory of insulin	10.4	16.7			33.3			0.0002
It is difficult to provide guidance and education on insulin injection to patients	16.9	23.3			55.5			0.0001
I do not have time to persuade patients to undergo insulin therapy or provide guidance on it	15.6	13.4			44.4			0.0003
It is difficult to educate staff (e.g., nurses) about insulin therapy	14.3	23.3			40.7			<.0001
*Consideration of burden on patients*								
It is difficult to recommend insulin therapy considering the pain associated with it	6.5			16.7			18.5	0.0196
The patient would have to pay more for treatment	76.6			53.3			62.9	0.1576
*Concerns regarding insulin therapy*								
Patients would resist insulin therapy	48.1			56.7			81.4	0.0005
I have concerns about the use of insulin therapy in elderly patients	38.1			43.3			81.5	<.0001
Hospitalization is necessary	5.2			6.7			22.2	0.0013
*Concerns regarding hypoglycemia*								
There is a higher risk of hypoglycemia with insulin therapy compared to other therapies	26.0			36.6			25.9	0.2168
My clinic/hospital is unable to provide treatment for hypoglycemia	0.0			16.6			25.9	<.0001
It is time-consuming for my clinic/hospital to provide treatment for hypoglycemia	3.9			10.0			22.2	0.0002
Patients would stop coming to my clinic/hospital if I recommended insulin therapy	2.6			10.0			11.1	0.0090
Compliance with insulin therapy tends to be low	5.2			3.3			0.0	0.1799

† P values were calculated by Kruskal-Walis test.

JDS-certified specialists exhibited less concern over insulin use than other physician groups. More than 30% differences in concerns between JDS-certified specialists and non-JDS-affiliated physicians were observed in the following items: “I’m not familiar with insulin therapy” (2.6% vs 37.0%, respectively), “It is difficult to select the type of insulin and adjust the dose” (3.9% vs 37.0%), “It is time-consuming to explain injection methods and the use of injection devices” (15.6% vs 51.8%), “I do not have staff (nurse, pharmacists) who can assist with explanations” (1.3% vs 55.5%), “It is difficult to provide guidance and education on insulin injection to patients” (16.9% vs 55.5%), “Patients would resist insulin therapy” (48.1% vs 81.4%), “I have concerns about the use of insulin therapy in elderly patients” (38.1% vs 81.5%).

A stepwise multiple linear regression analysis indicated four PAINT items as independent determinants which affect the rate of insulin use. A positive correlation was demonstrated in the following variables: “My clinic/hospital is not equipped to provide insulin therapy” (β = 0.334), “If necessary, I can refer the patients to a specialist” (β = 0.230), and “My clinic/hospital is unable to provide treatment for hypoglycemia” (β = 0.191). However, a negative correlation was observed in “I do not have time to persuade patients to undergo insulin therapy or provide guidance on it” (β = −0.257), (Table 3).

**Table 3:** Stepwise multiple linear regression analysis of the PAINT items associated with the rate of insulin use.

Variable (PAINT items)	Regression coefficient(B±SE; %)	Standardized partialregression coefficient (Beta)	Significance
My clinic/hospital is not equipped to provide insulin therapy	0.044±0.015	0.334	P = 0.0040
If necessary, I can refer the patients to a specialist	0.025±0.010	0.230	P = 0.0099
I do not have time to persuade patients to undergo insulintherapy or provide guidance on it	−0.029±0.010	−0.257	P = 0.0053
My clinic/hospital is unable to provide treatment forhypoglycemia	0.027±0.013	0.191	P = 0.0424

Dependent variable: rate of insulin use.

## Discussion

The present analyses provide important insights into current treatment preferences and physician barriers to insulin initiation in Japan. Our survey results of 134 Japanese physicians show that, in the management of diabetes, non-diabetes specialists have more concerns about insulin initiation than specialists, and that most of the concerns among non-specialists are related to practical burdens.

The data on the male/female ratio, age, BMI and HbA_1c_ values of the 11,656 patients with T2D surveyed in this study are similar to previously reported information on patients with T2D in Japan [21], [22]. Our data show that patients with T2D in Japan apparently have unsatisfactory control of HbA_1c_ with a mean of 7.5%. Approximately 20% of patients were still in suboptimal control (HbA_1c_ >8.4%).

Physician barriers to insulin initiation were suggested by an HbA_1c_ level at which physicians would initiate or initiated insulin. More than 90% of patients with HbA_1c_ level of >8.4% were not recommended insulin. The physicians stated that they would use insulin at an HbA1c level of 8.2% for themselves if they themselves required insulin. This value is close to an HbA1c level of 8.4% which was categorized as “unacceptable” in treatment guidelines including JDS and ADA, and therefore considered insufficiently high enough for insulin initiation. In other words, even physicians, who are supposed to aim at achievement of adequate glycemic control in the diabetes management, are not going to start insulin under the exposure of an insufficient HbA_1c_ level which can increase a risk of subsequent diabetic complications, and it is partially suggested that physicians underestimate the importance of achieving a targeted HbA_1c_ level recommended by the guidelines. Compared with this value, an HbA_1c_ level at which physicians responded they would recommend insulin to their patients have increased by 0.5% to 8.7%. An assumption of patient resistance for insulin initiation may be one possible explanation delaying an introduction of insulin therapy to the patients. Under this assumption, physicians may hesitate to introduce insulin therapy timely. In addition, the gap was observed between this HbA_1c_ value and actual value at which insulin therapy was recommended to patients. Patients were actually recommended insulin therapy at an HbA_1c_ level of 9.6%. This discordance is referred to as clinical inertia. Clinical inertia is defined as the recognition of a problem with a patient’s management but a failure to act [23]. Factors contributing to clinical inertia can include an assumption of patient resistance, actual resistance by the patients, failure to set clear target level, or insufficient communication with patients.

The result of PAINT showed physicians’ concerns over insulin use as potential factors contributing clinical inertia observed in this study. Non-JDS-affiliated physicians were particularly concerned about practical burdens related to giving explanation to the patient such as time required to explain and train patients to inject, difficulties explaining blood glucose monitoring and hypoglycemia management, or few trained support staff to guide patients. Preparing an explanatory material to facilitate explanations to patients may help reduce these burdens. In contrast, JDS-certified specialists reported insulin-related concerns less frequently overall. Problems related to complexity of insulin therapy such as determining insulin type and dose were not often observed, which may be due to sufficient experience of using insulin and comprehensive knowledge of diabetes and insulin in this group. The concerns most frequently expressed by the specialists were related to cost of therapy and use in the elderly. While there is less scope for overcoming barriers in JDS-certified specialists as practical barriers were rarely cited as a concern, specialists may benefit from an explanatory material to encourage patient acceptability of insulin since nearly 50% of specialists answered that patient would resist insulin therapy. An explanatory material specifically for elderly patients might be also useful. Although few concerns for insulin use were expressed by JDS-certified specialists, it should be noted that physicians may not introduce insulin therapy only with one single reason. Based on stepwise multiple linear regression analysis, four PAINT items were indicated as the predictive factors associating the lower rate of insulin use. Of these, the item “I do not have time to persuade patients to undergo insulin therapy or provide guidance on it” negatively correlated with the lower rate of insulin use. The one possible explanation is that physicians or other staff actually managed to give guidance or explanation to the patients if necessary even though physicians did not enough time. Or possibly, physicians who administrate insulin more frequently might perceive that they do not have enough time to give appropriate guidance.

While literature [16], [17], [24]–[27] frequently indicates physician barriers to insulin initiation, few studies have examined these barriers in detail. In the international DAWN study, it was reported that approximately 40% of physicians would delay insulin until absolutely necessary [14]. In a study of 157 family physicians in Israel, Nakar et al examined the reasons for non-initiation of insulin in patients meeting initiation criteria. More than 90% of physicians reported that patients would not comply with treatment, nearly 80% cited concerns over hypoglycemia, approximately 50% were worried that patients would not cope with the pain (of injecting or blood glucose measurements), and 47% were concerned about patients’ age [28]. Just 27% cited no experience with treatment as a barrier to initiation [28]. It is difficult to compare studies directly due to differences in design, question structure and cultural background; however, the physicians in the DAWN JAPAN study appeared less concerned about hypoglycemia, although both studies did identify that using insulin in elderly people was frequently a concern. In our study fewer than 5% of physicians were concerned about compliance with treatment.

There is a limitation that needs to be acknowledged and addressed regarding the present study. The limitation has to do with the extent to which physician barrier to insulin therapy actually delay the insulin initiation. The findings of this study did not show how strongly each factor would affect the initiation delay. Future assessments are necessary to address this issue.

In conclusion, our results show that physicians do have concerns about insulin use that may delay insulin initiation. As expected, non-specialists reported more concerns than specialists, even though both specialists and non-specialists appeared to delay insulin use frequently. Understanding physician barriers to insulin use is important to ensure that appropriate strategies can be employed to overcome these barriers.

## References

[1] UK Prospective Diabetes Study (UKPDS) Group (1998). Intensive blood-glucose control with sulphonylureas or insulin compared with conventional treatment and risk of complications in patients with type 2 diabetes (UKPDS 33).. Lancet.

[2] Shichiri M, Kishikawa H, Ohkubo Y, Wake N (2000). Long-term results of the Kumamoto Study on optimal diabetes control in type 2 diabetic patients.. Diabetes Care.

[3] Hunt LM, Valenzuela MA, Pugh JA (1997). NIDDM patients’ fears and hopes about insulin therapy: the basis of patient reluctance.. Diabetes Care.

[4] Lauritzen T, Scott A (2001). Barriers to insulin therapy in type 2 diabetes: a qualitative focus group research among patients, GPs and diabetologists.. European Union of General Practitioners.

[5] Wolfennbuttel BHR, Drossaert CHC, Visser AP (1993). Determinants of injecting insulin in elderly patients with type 2 diabetes mellitus.. Patient Educ Couns.

[6] Zambanini A, Newson RB, Feher M (1999). Injection related anxiety in insulin-treated diabetes.. Diabetes Res Clin Pract.

[7] Bogatean MP, Hâncu N (2004). People with type 2 diabetes facing the reality of starting insulin therapy: factors involved in psychological insulin resistance.. Practical Diabetes Int.

[8] Karter AJ, Subramanian U, Saha C, Crosson JC, Parker MM (2010). Barriers to insulin initiation: the translating research into action for diabetes insulin starts project.. Diabetes Care.

[9] Kunt T, Snoek FJ (2009). Barriers to insulin initiation and intensification and how to overcome them.. Int J Clin Pract.

[10] Hermanns N, Mahr M, Kulzer B, Skovlund SE, Haak T (2010). Barriers towards insulin therapy in type 2 diabetic patients: results of an observational longitudinal study.. Health Qual Life Outcomes.

[11] Gavin JR 3rd, Peragallo-Dittko V, Rodgers PT (2010). A new look at established therapies: practical tools for optimizing insulin use.. Diabetes Educ.

[12] U.K. Prospective Diabetes Study Group (2002). Sulfonylurea inadequacy: efficacy of addition of insulin over 6 years in patients with type 2 diabetes in the U.K. Prospective Diabetes Study (UKPDS 57).. Diabetes Care.

[13] Alberti G (2002). The DAWN (Diabetes Attitudes Wishes and Needs) study.. Pract Diab Int.

[14] International DAWN Advisory Panel (2005). Resistance to Insulin Therapy Among Patients and Providers: Results of the cross-national Diabetes Attitudes, Wishes, and Needs (DAWN) study.. Diabetes Care.

[15] Skovlund SE, Peyrot M (2005). The Diabetes Attitudes, Wishes, and Needs (DAWN) Program: A new approach to improving outcomes of diabetes care.. Diabetes Spectrum.

[16] Funnell MM (2007). Overcoming Barriers to the Initiation of Insulin Therapy.. Clinical Diabetes.

[17] Korytkowski M (2002). When oral agents fail: practical barriers to starting insulin.. International Journal of Obesity.

[18] World Medical Association (2000). Declaration of Helsinki: Recommendations Guiding Physicians in Biomedical Research Involving Human Subjects.. Amended by the 52nd General Assembly, Edinburgh, World Medical Association.

[19] Ministry of Education, Culture Sports, Science & Technology and the Ministry of Health, Labor & Welfare (2002). Ethical Guidelines for Epidemiological Research.. Tokyo: Ministry of Education, Culture, Sports, Science & Technology and the Ministry of Health, Labor & Welfare.

[20] Japan Diabetes Clinical Data Management Study Group (2006). The status of diabetes control and antidiabetic drug therapy in Japan – A cross-sectional survey of 17,000 patients with diabetes mellitus (JDDM1). Diab. Res. Clin.. Pract.

[21] Committee of the Japan Diabetes Society on the Diagnostic Criteria of Diabetes Mellitus (2010). Report of the Committee on the Classification and Diagnostic Criteria of Diabetes Mellitus.. Journal of Diabetes Investigation.

[22] Ministry of Welfare, Japan (2002). Report of national survey of Diabetes..

[23] Phillips LS, Branch WT, Cook CB, Doyle JP, El-Kebbi IM (2001). Clinical inertia.. Ann Intern Med.

[24] Meece J (2006). Dispelling myths and removing barriers about insulin in type 2 diabetes.. Diabetes Educ.

[25] Cuddihy RM, Philis-Tsimikas A, Nazeri A (2011). Type 2 diabetes care and insulin intensification: is a more multidisciplinary approach needed? Results from the MODIFY survey.. Diabetes Educ.

[26] Haque M, Emerson SH, Dennison CR, Navsa M, Levitt NS (2005). Barriers to initiating insulin therapy in patients with type 2 diabetes mellitus in public-sector primary health care centres in Cape Town.. S Afr Med J.

[27] Peyrot M, Rubin RR, Khunti K (2010). Addressing barriers to initiation of insulin in patients with type 2 diabetes.. Prim Care Diabetes.

[28] Nakar S, Yitzhaki G, Rosenberg R, Vinker S (2007). Transition to insulin in Type 2 diabetes: family physicians' misconception of patients' fears contributes to existing barriers.. J Diabetes Complications.

